# Case report: Effectiveness of Janus kinase inhibitors in the management of isolated noninfectious uveitis: a case series

**DOI:** 10.3389/fphar.2025.1509404

**Published:** 2025-03-06

**Authors:** Nina Vidic Krhlikar, Matija Tomšič, Polona Jaki Mekjavić, Pia Klobučar, Nataša Vidović Valentinčič

**Affiliations:** ^1^ Eye Hospital, University Medical Centre Ljubljana, Ljubljana, Slovenia; ^2^ Department of Rheumatology, University Medical Centre Ljubljana, Ljubljana, Slovenia; ^3^ Faculty of Medicine, University of Ljubljana, Ljubljana, Slovenia; ^4^ Jožef Stefan Institute, Ljubljana, Slovenia

**Keywords:** Janus kinase inhibitors, noninfectious uveitis, baricitinib, upadacitinib, posterior uveitis

## Abstract

**Purpose:**

This study aimed to assess the efficacy of Janus kinase inhibitors (JAK-i) in the treatment of refractory isolated non-infectious uveitis.

**Case presentation:**

We examined a case series involving three patients with isolated non-infectious uveitis, who were managed between December 2019 and December 2023 at The Eye Hospital, University Medical Centre Ljubljana. The JAK-i therapy was initiated due to the patients’ unresponsiveness to disease-modifying antirheumatic drugs.

**Outcomes:**

In our case series, two patients presented with anterior and intermediate uveitis, one with posterior uveitis. None of the patients had any associated systemic disease. After the initiation of JAK-I therapy, all the patients achieved remission lasting for more than 1 year. No significant side effects were observed in any of the patients throughout a mean follow-up period of 31.6 months (range, 16–55 months).

**Conclusion:**

In this report, we present three cases of refractory isolated non-infectious uveitis successfully treated with JAK-i. This is the first report on the use of baricitinib and upadacitinib in this context. Our findings suggest that alternative use of JAK-i, apart from tofacitinib alone, may be an effective treatment option for such patients.

## 1 Introduction

Uveitis refers to inflammation of the uvea, which includes the iris, ciliary body, and choroid; however, it can affect any part of the eye. Uveitis can be infectious or noninfectious and is categorized into anterio^4^r, intermediate, posterior, and panuveitis based on the primary site of inflammation. Symptoms of uveitis range from pain and redness to vision loss. Uveitis is often idiopathic but can be associated with systemic inflammatory conditions, such as HLA-B27-related diseases, sarcoidosis, and Behcet’s disease, or can occur in isolation—limited to a specific anatomical part of the eye (McCluskey and Powell, 2004; Jabs et al., 2005; Generali et al., 2015). Complications of uveitis may pose significant risks to both vision and the eye itself (McCluskey and Powell, 2004; Generali et al., 2015).

First-line treatments for both systemic-associated and isolated noninfectious uveitis (NIU) typically include corticosteroids. However, long-term use of corticosteroids is restricted due to their potential side effects. In cases where patients are resistant to corticosteroids or have severe, vision-threatening uveitis, the use of disease-modifying antirheumatic drugs (DMARDs) may be necessary (Dick et al., 2018).

The Janus kinase (JAK) pathway plays a pivotal role in the regulation of inflammatory cells by transmitting signals from cytokines and growth factors. Dysregulation of the JAK pathway significantly contributes to the pathogenesis of diverse inflammatory and autoimmune disorders (Pleyer et al., 2017; Fragoulis et al., 2019; Sharma et al., 2023).

The use of Janus kinase inhibitors (JAK-i) in the treatment of isolated NIU is a novel approach; hence, there is limited data on the subject (Paley et al., 2019; Liu et al., 2021). Current ongoing studies aim to assess the safety and efficacy of two JAK-I in patients with NIU: HUMBOLDT (phase III) study for filgotinib (Srivastava et al., 2024) and NEPTUNE (phase II) study for brepocitinib. (Iapoce, 2024). Both studies have shown promising results; however, the HUMBOLT study was terminated prematurely after 24 weeks due to business reasons, eventhough not enough safety and efficacy information had been collected.

In this study, we examined three cases of isolated NIU that were all successfully treated using JAK-i. Importantly, to the best of our knowledge, this is the first study to document the use of baricitinib and upadacitinib in the management of isolated NIU.

## 2 Outcomes

We examined three consecutive cases of patients with isolated NIU from 2019 to 2023 who were managed at the Eye Hospital of the University Medical Centre of Ljubljana, a tertiary-care academic medical institution in Slovenia. These patients were treated with JAK-i due to refractory inflammation, despite prior treatment with corticosteroids and DMARDs during the observation period. All JAK inhibitors were given orally. Both drugs are “off label” for the treatment of uveitis. Although the literature provides some data on the efficacy of tofacitinib in the treatment of uveitis, patients have preferred baricitinib because it is dosed only once daily.

The diagnosis of uveitis was made according to the Standardization of Uveitis Nomenclature (SUN) Working Group criteria. Disease activity was assessed using the same criteria (Jabs et al., 2005).

All patients underwent a complete ophthalmic examination, including the assessment of best corrected visual acuity (BCVA), intraocular pressure (IOP), slit lamp biomicroscopy, dilated fundoscopy, angiography (with fluorescein (FA) and indocyanine-green (ICG) dye), and optical coherence tomography (OCT). Extensive workup by ophthalmologists and rheumatologists was done to rule out specific autoimmune, infectious, rheumatological, paraneoplastic, and neoplastic causes of inflammation.

## 3 Case series

Between 2019 and 2023, we treated three patients diagnosed with standard therapy-resistant NIU with JAK-i. Details of the patients’ clinical data are presented in Table 1. The mean time to full remission was 6.3 months (range, 4–9 months), with a mean follow-up duration of 31.6 months (range, 16–55 months). The mean best corrected visual acuity (BCVA) before JAK-i treatment was 04 (Snellen), and this improved to 0.6 after JAK-i treatment. None of our patients developed serious side effects; however, one patient had a slight temporary increase in liver transaminase levels.

**TABLE 1:** Clinical data and a timeline from the episode of care of patients with uveitis treated with JAK-i.

	Case 1	Case 2	Case 3
Sex	F	M	M
Age at presentation (years)	28	41	59
Diagnosis/year	Anterior uveitis and intermediate uveitis/2021	Anterior uveitis and intermediate uveitis/2013	Posterior uveitis (ampiginous choroiditis)/2018
Laterality	Bilateral	Bilateral	Bilateral
Systemic therapy before JAK-i	MP, MMF, ADA, TCR	MP, MTX, MMF, CsA, ADA	MP, MMF, ADA
BCVA before JAK-i treatment	RE: 0.02/LE: 0.2	RE: 0.6/LE: 1.0	RE: 0.4/LE: 0.6
Time from presentation to JAK-i treatment (months)	1 y 4 m	6 y 5 m	3 y 1 m
Indication for JAK-I treatment	PSTF	PSTF	PSTF
Type of JAK-i	BRC	BRC, UPDC	BRC
Concomitant treatment at the start of JAK-i/at the final visit	32/0 mg MP alternating doses every day	12/0 mg MP alternating doses every day	32/4 mg MP alternating doses every day
Time to remission	4 m	6 m	9 m
Follow up time	1 y 11 m	3 y 7 m	1 y 5 m
Adverse events - transient	Rise in ALT: 0,65 μkat/L (NR: 0–0.56 μkat/L)	—	—
Surgical procedure	Cataract surgery RE	Cataract surgery RE and LE TREC LE	—
BCVA at the final visit	RE: 0.8/LE: 0.1	RE: 1.0/LE: 1.0	RE: 0.4/LE: 0.5

Legend: BCVA, best corrected visual acuity; F, female; M, male; LE, left eye; RE, right eye; m, months; w, weeks; y, years; PSTF, Previous systemic treatment failure; NR, normal range; MP, methylprednisolone; ADA, adalimumab; BRC, baricitinib; CsA–cyclosporine; JAK-i, Janus kinase inhibitors; MMF, mofetil mycophenolate; MTX, methotrexate; TCR, tacrolimus; BRC, baricitinib; UPDC, upadacitinib; TREC, trabeculectomy.

## 4 Case 1

### 4.1 Patient information and clinical findings

**FIGURE 1:**
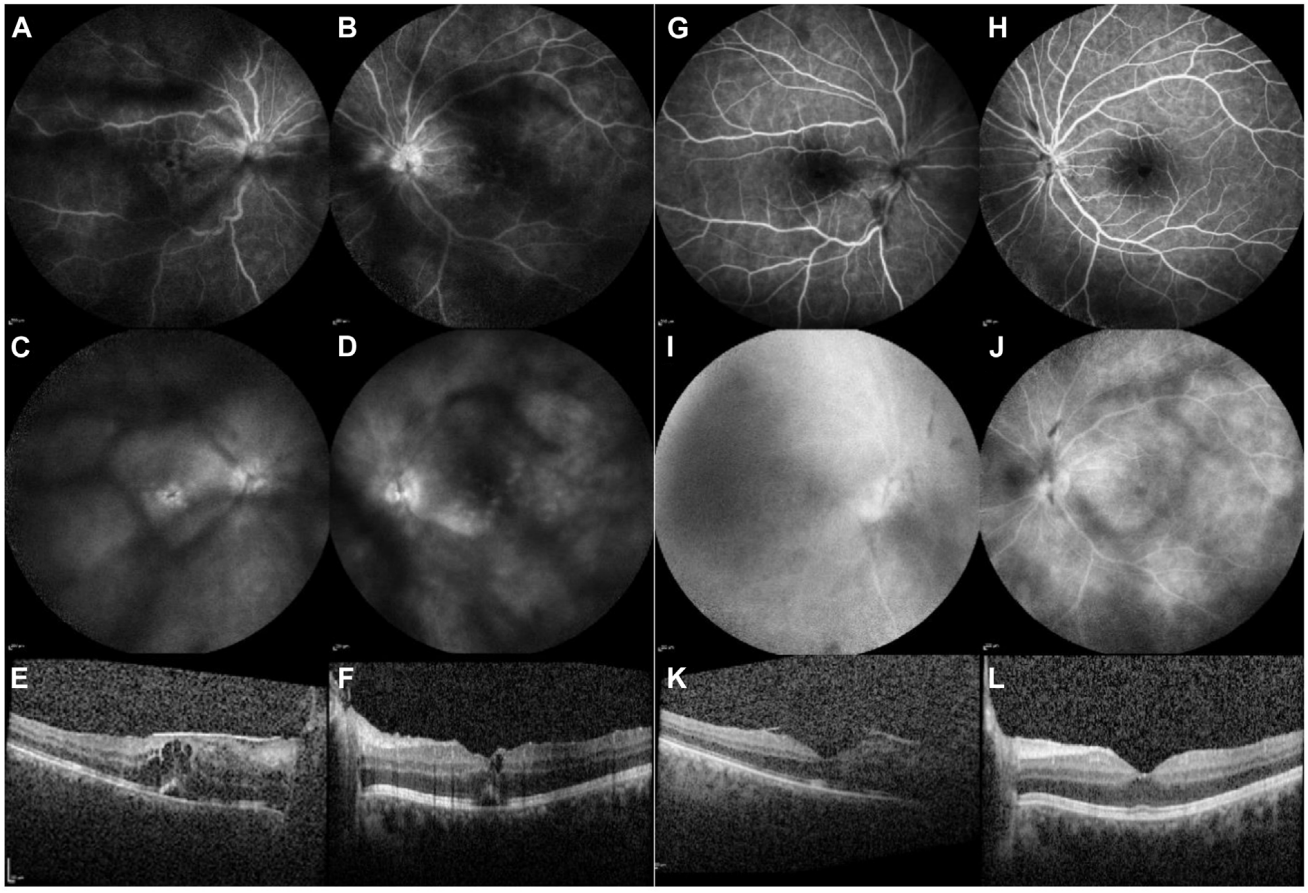
Case 1. Imaging before the initiation of baricitinib therapy [left column: **(A–F)**] and 4 months thereafter commencement of treatment [right column: **(G–L)**]. FA of the RE and LE [early frames: **(A, B)**; late frames: **(C, D)**]: before baricitinib treatment, visualisation are obscured due to vitreous opacifications. Peripapillary and retinal vascular leakage are also observed, along with pooling in the macula indicative of CME. Four months after the initiation of baricitinib treatment [early frames: **(G, H)**; late frames: **(I, J)**], reduced peripapillary and retinal vascular leakage can be observed, and no pooling in the macula is evident with FA. OCT of the macula before the initiation of baricitinib treatment [right eye: **(E)**, and left eye: **(F)**] shows intraretinal and subretinal fluid. Four months after the initiation of baricitinib treatment, a reduction of CME is observed in the RE **(I)** and a complete resolution of CME is seen in the LE **(J)**.

In 2021, a 28-year-old woman presented with a 5-month history of blurred vision. Her BCVA in both eyes was 0.3. Slit-lamp examination revealed 3+ aqueous and vitreous cells. OCT and FA revealed cystoid macular edema (CME), more pronounced in the right eye (RE) (Figures 1A–F). A diagnosis of bilateral NIU–anterior and intermediate uveitis with CME was made according to SUN criteria (Jabs et al., 2005).

### 4.2 Timeline

Data in row 1, Table 1. Treatment commenced with intravenous boluses of 500 mg of MP daily for three consecutive days, followed by oral MP at an initial dose of 48 mg daily, which was gradually tapered to 12 mg daily. Subsequently, multiple DMARDs (MP, MMF, ADA, and TCR) were added, leaving the patient with a BCVA of 0.02 in the RE and 0.2 in the left eye (LE). After 1 year and 4 months, systemic therapy with 4 mg of baricitinib daily was initiated, with tapering doses of MP only. Patient stayed on MP 4 mg every other day. Four months later, complete resolution of both intraretinal and subretinal fluid was observed (Figures 1G–L). The patient’s BCVA improved from 0.02 to 0.3 in the RE and from 0.2 to 0.4 in the LE. Exudation in the anterior chamber (AC) and vitreous were reduced to less than 0.5+ cells.

Subsequently, cataract surgery was performed on the RE, resulting in an improvement of BCVA to 1.0.

### 4.3 Outcomes and follow up

During the 23-month follow-up period after the commencement of JAK-i therapy, the patient remained stable, and her BCVA at the last follow-up visit was 0.8 in the RE and 0.1 in the LE. The reduction in BCVA was attributed to posterior capsular opacification in the RE and cataract in the LE. Throughout the follow-up period, the patient experienced only a slight elevation in alanine aminotransaminase (ALT) levels, measured at 0.65 μkat/L (≤0.56 μkat/L).

## 5 Case 2

### 5.1 Patient information and clinical findings

**FIGURE 2:**
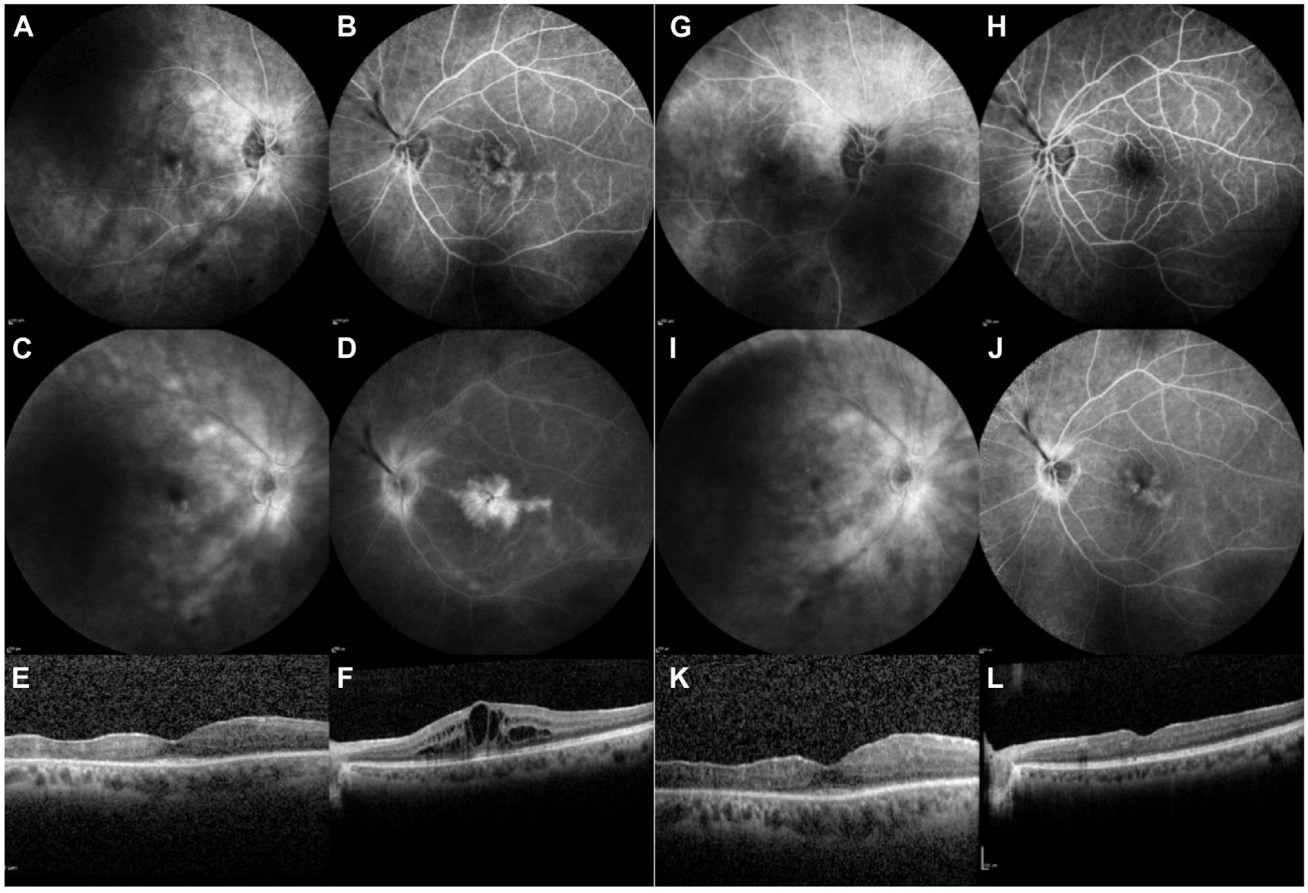
Case 2. Imaging during treatment with baricitinib, before starting treatment with upadacitinib (left).[column: **(A–F)**] and 14 months thereafter [right column: **(G–L)**]. FA of the RE and LE [early frames: **(A, B)**; late frames: **(C, D)**]: shows bilateral peripapillary and retinal vascular contrast leakage, with mild pooling in the right, and is very pronounced in the left macula due to CME **(E, F)**. Fourteen months after the initiation of upadacitinib treatment, less peripapillary and retinal vascular leakage are observed, and no pooling in the macula is evident [early frames: **(G, H)**; late frames: **(I, J)**]. OCT of the macula shows CME in the LE during baricitinib treatment **(F)**, with resolution of CME observed 14 months after the initiation of upadacitinib treatment **(L)**.

In 2013, a 41-year-old man, previously diagnosed with chronic bilateral NIU–anterior and intermediate uveitis, presented with diminished BCVA of 0.3 in the RE and 0.9 in the LE. Slit-lamp examination revealed no cells in the AC and significant vitritis in both eyes, with 3+ vitreous cells in the RE and 1+ vitreous cells in the LE. FA revealed bilateral peripapillary and retinal vascular contrast leakage, while OCT showed CME in the RE (Figures 2A–F). A diagnosis of bilateral intermediate uveitis was made according to SUN criteria (Jabs et al., 2005).

### 5.2 Timeline

Data in row 2, Table 1. Treatment commenced with a 3-day course of intravenous MP at a dose of 500 mg daily, followed by multiple DMARDs (MP, MTX, MMF, CsA, and ADA). Additionally, several dexamethasone intravitreal implants were administered in the LE due to chronic CME. While remission of uveitis was achieved, the patient developed secondary glaucoma and cataracts.

Trabeculectomy was performed in the LE to stabilize IOP, followed by cataract surgery in the LE and subsequently in the RE. The patient has a recurrence of uveitis after 6 years and 5 months of treatment and was switched to 4 mg of baricitinib daily; however, the CME in the LE persisted. FA performed 1 year after the initiation of baricitinib still revealed active inflammation; therefore, baricitinib was replaced with 15 mg of upadacitinib daily. Only the MP dose was tapered and has now been stopped completely. Six months after commencing upadacitinib therapy, resolution of inflammation, particularly in terms of exudation, was observed, including improvement in FA and OCT findings (Figures 2G–L). However, regular dexamethasone implants for chronic CME were still required for the LE.

### 5.3 Outcomesectionbs and follow up

After 3 years and 7 months of upadacitinib therapy, no relapse of uveitis occurred, and the patient’s visual acuity improved to 0.9 in the RE and 1.0 in the LE. Throughout the treatment course, the patient experienced no adverse events.

## 6 Case 3

### 6.1 Patient information and clinical findings

**FIGURE 3:**
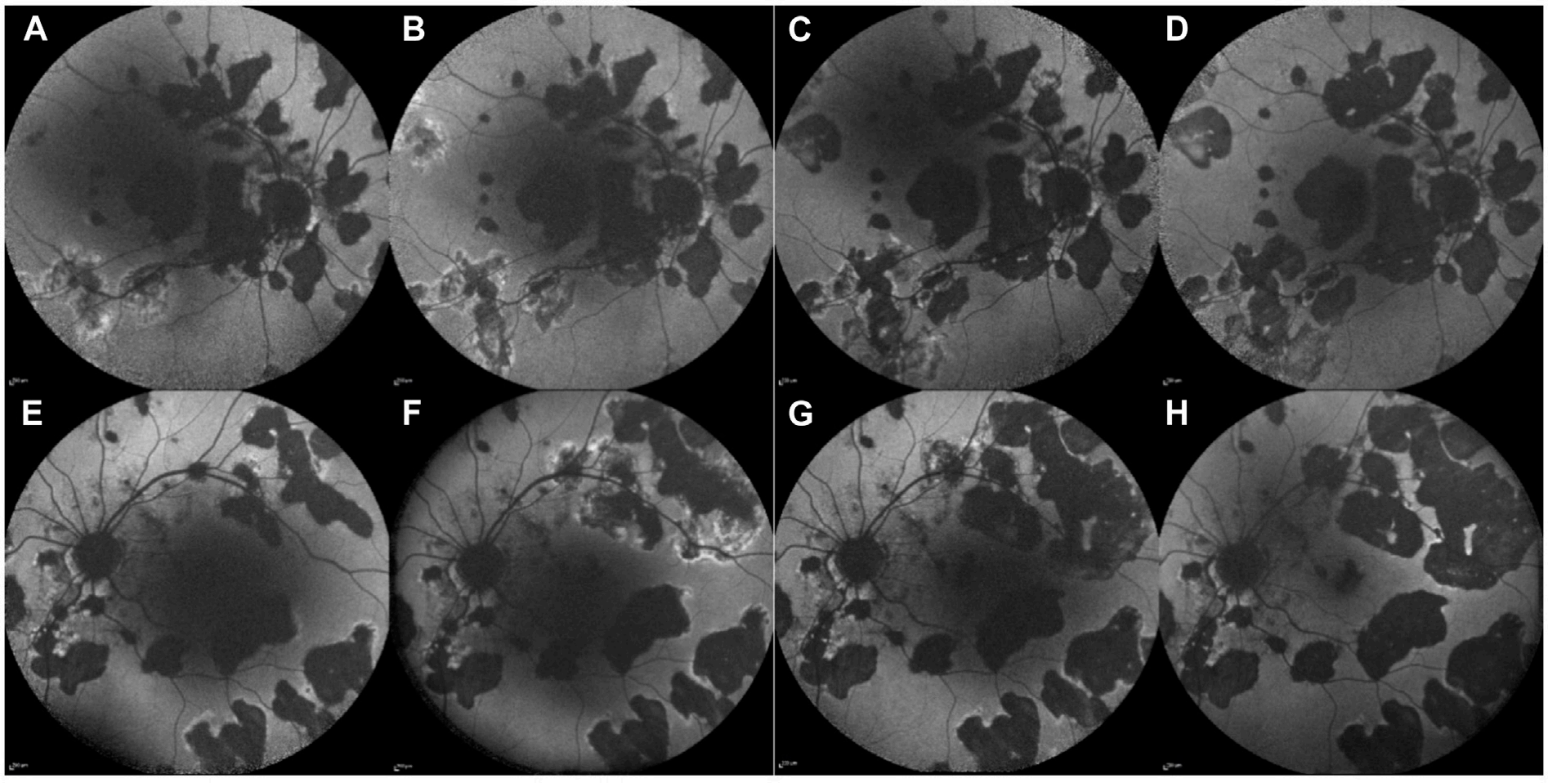
Case 3. Fundus autofluorescence (FAF) of the RE (upper line) and LE (lower line) before JAK-i therapy **(A, B, E, F)** and after the initiation of JAK-i therapy **(C, D, G, H)**. FAF images taken over 1 year **(A, E)** and 6 months **(B, F)** before the initiation of JAK-i therapy reveals numerous hypo-autofluorescent lesions with irregular hyper-autofluorescent borders, which increase in size and number over time. FAF images taken 3 months **(C, G)** and 9 months **(D, H)** after the initiation of JAK-i treatment show a reduction of the hyper-autofluorescent border and no new lesions.

In 2018, a 59-year-old man presented with a 1-month history of decreased visual acuity and increased floaters in the LE, with a BCVA of 0.02 in the LE and 0.9 in the RE. The diagnosis of posterior uveitis was established. Slit-lamp examination revealed granulomatous endothelial precipitates, 0.5+ cells in the AC, and 2+ vitreous cells in both eyes. Both optic discs appeared hyperemic, and yellowish inflammatory retinal lesions were observed, more pronounced in the LE, with some peripheral pigmented atrophic lesions in the mid-periphery in both eyes, corresponding to irregular hyper-autofluorescent or hypo-autofluorescent lesions with an irregular hyper-autofluorescent border on fundus autofluorescence (FAF) (Figures 3A, B, E, F). FA displayed multiple early-phase hypofluorescence and peripapillary hyperfluorescence. Indocyanine green angiography (ICGA) showed corresponding hyperfluorescence. OCT revealed a peripapillary hyperreflective lesion above the retinal surface with surrounding edema. Owing to the above findings, the patient was diagnosed with ampiginous choroiditis.

### 6.2 Timeline

Data in row 3, Table 1. Treatment began with IV boluses of 500 mg of MP daily for three consecutive days, followed by oral MP at an initial dose of 48 mg daily, and was gradually tapered to 12 mg, with the addition of various DMARDs (MP, MMF, and adalimumab). Peripapillary choroidal neovascularization (CNV) that developed in the RE before the administration of adalimumab (ADA) required multiple intravitreal anti vascular endothelial growth factor (VEGF) injections. Despite systemic treatment, inflammation persisted, and the retinal lesions increased in size, with new lesions appearing over the course of 6 months. After 3 years, ADA was replaced with 4 mg of baricitinib daily. Only the MP dose was tapered and has now been stopped completely. Nine months after commencing baricitinib the inflammation. stabilized in terms of new chorioretinal lesions – no new ones were documented.

### 6.3 Outcomes and follow up

No additional chorioretinal lesions or systemic side effects were documented during the 17-month follow-up period (Figures 3C, G after 3 months of treatment with baricitinib; Figures 3D, H after 9 months of treatment with baricitinib). His last documented BCVA on his RE was 0.4 and LE 0.5.

## 7 Discussion

Managing refractory non-infectious uveitis remains challenging despite advancements in disease understanding, diagnostics, and DMARD use. Experimental uveitic models have demonstrated that JAK inhibitors, such as tofacitinib and upadacitinib, can modulate pro-inflammatory T cells and reduce cytokine expression, making them promising options for the treatment of uveitis (Amadi-Obi et al., 2007; Luger et al., 2008; Stevenson et al., 2014; Huang et al., 2023). However, studies on the use of JAK-i, especially in the management of isolated ocular inflammation, are limited.

Previous studies on the use of JAK-i in treating isolated NIU include a retrospective cohort study and two case reports of three patients who presented with bilateral NIU; all the patients were treated with tofacitinib due to previous treatment failures in two cases and treatment intolerance in one case. One of the patients received concurrent therapy with methotrexate (MTX), while the other two patients received MP 5 mg/day, with the addition of MTX in one case. Successful control of inflammation was achieved in all three patients, and no relapses or adverse events were reported during a mean follow-up of 6 months (range, 1–11 months) (Paley et al., 2019; Liu et al., 2021; Sharma et al., 2023).

In this study, all our patients underwent a complex treatment regimen involving corticosteroids and multiple DMARDs. In all three patients, baricitinib therapy was initiated, for the first time in patients with NIU, resulting in the remission of ocular inflammation in two of our patients. In one patient of intermediate uveitis, baricitinib was later switched to upadacitinib, resulting in effective inflammation control. The mean follow-up duration for our patients was 31.6 months (range, 16–55 months), which is the longest reported follow-up duration for NIU so far.

The primary safety data on JAK-i are derived from the ORAL Surveillance (ORALSURV) study (Winthrop and Cohen, 2022). The study evaluated the use of tofacitinib in the treatment of rheumatoid arthritis (RA) and showed that JAK-i treatment had a similar risk of serious infections and major adverse cardiovascular events when compared to other biologic therapies, but was associated with a higher risk of malignancy. In addition, tofacitinib treatment in patients with RA was associated with a dose-dependent risk of venous thromboembolism (VTE). In a recently published case report, a middle-aged woman with systemic sclerosis was treated with flingotinib and developed central retinal vein occlusion and ciliary artery occlusion in her right eye, resulting in severe, irreparable vision loss (Jevnikar et al., 2024). This underscores the need to assess the specific risk-benefit profile, specifically in patients with predispositions to VTE or malignancy and administration of JAKis should be made with caution in patients with an increased cardiovasular risk Other reported side effects of JAK-i include leucopenia, anemia, alterations in lipid profile, and elevated liver transaminases. However, these side effects are usually transient (Schwartz et al., 2017; Fragoulis et al., 2019). Only a slight increase in liver transaminase levels was observed in one of the patients in our study.

This study has some limitations, including the small sample size and its retrospective design. However, no prior reports have documented the use of upadacitinib and baricitinib in treating isolated NIU, nor have there been follow-up studies of JAK inhibitor treatment extending beyond 11 months except in the HUMBOLDT study, which was terminated prematurely.

## 8 Conclusion

Our case series suggests that JAK-i, apart from tofacitinib, could be an effective treatment option for the management of refractory NIU. Although our study’s sample size was small, our follow-up duration of 2.5 years highlights the possible safety and long-term efficacy of JAK-i. Nevertheless, more controlled trials are needed, and vigilant patient monitoring is required.

## Data Availability

The original contributions presented in the study are included in the article/supplementary material, further inquiries can be directed to the corresponding author.
